# Editorial: The Impact of Social Connections on Patients' Health

**DOI:** 10.3389/fpsyg.2022.909498

**Published:** 2022-04-28

**Authors:** Davide Mazzoni, Elizabeth A. Baker, Darrell L. Hudson, Gabriella Pravettoni

**Affiliations:** ^1^Department of Oncology and Hemato-Oncology, University of Milan, Milan, Italy; ^2^Behavioral Science and Health Education, College for Public Health and Social Justice, Saint Louis University, St. Louis, MO, United States; ^3^Brown School at Washington University in St. Louis, St. Louis, MO, United States; ^4^Applied Research Division for Cognitive and Psychological Science, IEO, European Institute of Oncology IRCCS, Milan, Italy

**Keywords:** social support, health, social connections, patients, cancer, lupus

As social connections are intrinsic to the concept of person (van Zomeren, 2016), they are also a fundamental aspect of a patients' life. Moreover, being a patient implies the presence of at least one factor whether a disease or health condition, that can make a patient vulnerable and in need of a supportive and empowering relationships. The support received from social connctions is critical to helping people to live a meaningful life, even moreso among people who are managing complex diseases and health conditions (Mazzoni et al., 2014; Bailo et al., 2019). In this sense, social connections are one of the most powerful modifiable factors at our disposal for promoting the physical and psychological health of patients (Cassel, 1976).

The scientific challenge is thus to better understand, from a theory-based and evidence-based perspective, under which conditions social connections are really beneficial, meeting patients' needs. Adopting such a perspective, the guest editors and the authors joined together their multidisciplinary contributions to provide a scientific journey across different diseases and approaches.

In this regard, not all social connections are functional and from a theoretical point of view, the opinion article by Sebri et al. argues that unsupportive social relationships negatively affect one's self-representation, an important issue for health management in breast cancer patients (“Injured Self”; Sebri et al., 2020).

In a similar line of thought, the work by Izydorczyk et al., focuses on postpartum women with diastasis recti abdominis (DRAM), to investigate the relationship between perceived social support from the partner, family and friends, and their body image. Results showed that social support of partners, family, and friends are the predictors of self-assessment of general body appearance. Social support from family is a predictor of self-assessment of the health of the body. Social support from friends is a predictor of self-esteem regarding weight and fear of gaining weight.

Moreover, the study by Kazukauskiene et al. aims to examine the associations between perceived social support and subjective fatigue levels in individuals with coronary artery disease, with and without depressive symptoms. The study provides a novel insight into understanding the relationship between support, depression and fatigue, which differs in groups of individuals with and without coronary artery disease.

Some contributions of this Research Topic are particularly relevant for developing interventions for cancer patients. In this regard, the research protocol by Marzorati et al. focuses on a possible intervention that aims to manage and prevent psychological problems related to social isolation in cancer patients.

The contribution by Durosini et al. presents sports-based interventions for improving the health and quality of life of 80 female cancer survivors, with the idea that through some sports activities participants could develop meaningful social relationships that affect their wellbeing.

In the case of Systemic Lupus Erythematosus (SLE) the literature has largely demonstrated the importance of social connections (Mazzoni and Cicognani, 2016; Leung et al., 2019). The study by White et al. presents three methodologies, respectively, peer-to-peer, patient support group, and a patient navigator program that were implemented among largely African American women with SLE at the Medical University of South Carolina (MUSC).

Finally, the study by Muhorakeye and Biracyaza explores the barriers to mental health services utilization, with the idea that identifying the barriers to the utilization of mental health services, might contribute to mitigating them. For example, stigmatization within social networks was reported as one of the main barriers to seeking mental health care, therefore it is critical to address stigma within community members in order to increase empathy and improve service use.

In sum, this Research Topic offers insight into the complex processes linking social connections and patients' health, investing such processes in specific contexts and diseases. All contributions also identified several limitations s in the current knowledge in this field, proposing innovative lines of research to fill such gaps. More research and practice needs to be conducted in order to gain a fuller understanding of the real impact that social connections play in the patients' lives and how researchers and practioners can intervene. Finally, as Guest Editors, we hope that these contributions will be inspiring for the health professionals, who every day put their efforts into building supportive networks, that make the patients feel heard and validated and able to express their own potential for an effective partnership in the disease management.

## Author Contributions

DM, EB, and DH wrote sections of the manuscript. All authors contributed to conception of the editorial, manuscript revision, read, and approved the submitted version.

## Funding

This work was partially supported by the Italian Ministry of Health with Ricerca Corrente and 5 × 1000 funds for IEO European Institute of Oncology IRCCS.

## Conflict of Interest

The authors declare that the research was conducted in the absence of any commercial or financial relationships that could be construed as a potential conflict of interest.

## Publisher's Note

All claims expressed in this article are solely those of the authors and do not necessarily represent those of their affiliated organizations, or those of the publisher, the editors and the reviewers. Any product that may be evaluated in this article, or claim that may be made by its manufacturer, is not guaranteed or endorsed by the publisher.
